# Interaction between Bluetongue virus outer capsid protein VP2 and vimentin is necessary for virus egress

**DOI:** 10.1186/1743-422X-4-7

**Published:** 2007-01-15

**Authors:** Bishnupriya Bhattacharya, Rob J Noad, Polly Roy

**Affiliations:** 1 Department of Infectious and Tropical Diseases, London School of Hygiene and Tropical Medicine, Keppel Street, London, WC1E 7HT, UK

## Abstract

**Background:**

The VP2 outer capsid protein Bluetongue Virus (BTV) is responsible for receptor binding, haemagglutination and eliciting host-specific immunity. However, the assembly of this outer capsid protein on the transcriptionally active viral core would block transcription of the virus. Thus assembly of the outer capsid on the core particle must be a tightly controlled process during virus maturation. Earlier studies have detected mature virus particles associated with intermediate filaments in virus infected cells but the viral determinant for this association and the effect of disrupting intermediate filaments on virus assembly and release are unknown.

**Results:**

In this study it is demonstrated that BTV VP2 associates with vimentin in both virus infected cells and in the absence of other viral proteins. Further, the determinants of vimentin localisation are mapped to the N-terminus of the protein and deletions of aminio acids between residues 65 and 114 are shown to disrupt VP2-vimentin association. Site directed mutation also reveals that amino acid residues Gly 70 and Val 72 are important in the VP2-vimentin association. Mutation of these amino acids resulted in a soluble VP2 capable of forming trimeric structures similar to unmodified protein that no longer associated with vimentin. Furthermore, pharmacological disruption of intermediate filaments, either directly or indirectly through the disruption of the microtubule network, inhibited virus release from BTV infected cells.

**Conclusion:**

The principal findings of the research are that the association of mature BTV particles with intermediate filaments are driven by the interaction of VP2 with vimentin and that this interaction contributes to virus egress. Furthermore, i) the N-terminal 118 amino acids of VP2 are sufficient to confer vimentin interaction. ii) Deletion of amino acids 65–114 or mutation of amino acids 70–72 to DVD abrogates vimentin association. iii) Finally, disruption of vimentin structures results in an increase in cell associated BTV and a reduction in the amount of released virus from infected cells.

## Background

**Figure 1:**
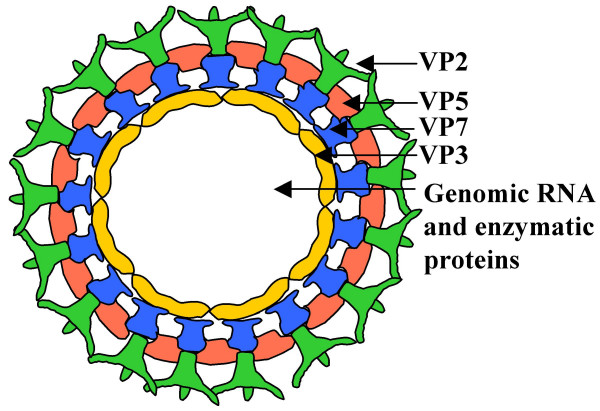
**Schematic of the mature BTV particle**. Organisation of the major structural proteins VP2, VP5, VP3 and VP7 in the architecturally complex BTV particle. On entry into cells the outer capsid proteins VP2 and VP5 are lost, releasing a transcriptionally active core particle.

Bluetongue virus (BTV), an insect (*Culicoides sp*) transmitted double-layered non-enveloped virus, is the type species virus of the genus *Orbivirus*, in the family *Reoviridae*. BTV is an economically important virus and causes hemorrhagic disease in sheep and other ruminants [1]. The virus has a genome consisting of ten double stranded (ds) RNA segments, located in a core particle made up of two major proteins (VP3 and VP7) and three minor proteins (VP1, VP4 and VP6). In the mature particle, this core is coated by two outer capsid proteins, VP2 (110 kDa) and VP5 (60 kDa) (see schematic, Fig. 1). The most exposed protein on the mature virion, VP2, is responsible for receptor binding [2-4], haemagglutinating activity [5] and elicits host specific immunity [6-8]. The smaller protein, VP5, is involved in cell penetration during the initial stages of infection [9,10]. After entry into cells, the virus is uncoated by removal of VP2 and VP5 to yield the core particle that is transcriptionally active. This complex is an end-point in virus disassembly and protects the viral dsRNA genome from intracellular surveillance mechanisms.

Like other members of *Reoviridae*, BTV replicates in the cytoplasm of infected cells. Electron microscopic analysis of thin sections of BTV infected cells have revealed a large numbers of virus-specific tubules, and juxtanuclear inclusions bodies containing virus-like particles in addition to small numbers of intracellular virus particles (15). While the tubules are multimers of NS1 [11], inclusion bodies are predominantly formed by NS2 [12,13]. Similar to rotavirus inclusion bodies [14], it has been speculated that during BTV replication and assembly NS2 binds core proteins, viral single-stranded (ssRNA) [15,16] and recruits these components to the inclusion bodies [17]. Additionally, it has been hypothesised that the viral genome interacts with the minor structural proteins to form the transcriptase complex which is subsequently encapsulated by a single shell of VP3 to form the subcore of the assembling virus [18]. The subcore acts as a scaffold for the deposition of VP7 trimers, thus forming a stable core structure [19-21]. However, the outer capsid proteins, VP2 and VP5 have not been localised to the viral inclusion bodies and it is still not clear when and where in the cell are they assembled on to the core. As the outer capsid proteins, particularly VP2, would effectively block the channels in the core that are used during transcription of the viral genome it is unlikely that the coating of cores would be an uncontrolled process.

Previous electron microscopy studies have indicated that BTV particles can be found attached to vimentin intermediate filaments [22]. Negative staining of intact, infected cells labelled with anti-VP2 antibody revealed that BTV particles were also present under the cell membrane as well as on the cell surface [23]. In addition, both virus aggregates and single virion particles retain an association with the cortical layer of the cytoskeleton following cell lysis. This has lead to speculation that there may be interaction of the particle with the actin-rich cortical layer underlying the cell membrane [23]. Recent studies have shown that NS3, the only virus-coded glycoprotein of BTV, interacts with VP2 as well as Tsg101 and the p11 subunit of the heterotetrameric calpactin II complex, thereby facilitating virus release [24,25]. Thus, in addition to its role in virus attachment, VP2 is emerging as a key player in the control of BTV assembly and egress from infected cells. A detailed understanding of the control of VP2 association with the newly assembled core is therefore an important step in understanding virus assembly and egress.

In this manuscript we demonstrate that normal subcellular distribution of VP2 relies on an association with vimentin intermediate filaments, and identify key residues in this interaction located in the N-terminus of the protein. Furthermore, disruption of the vimentin filament network using pharmacological inhibitors lead to a disruption of virus egress.

## Results

### In infected cells VP2 localises to punctuate areas within the cytoplasm

**Figure 2:**
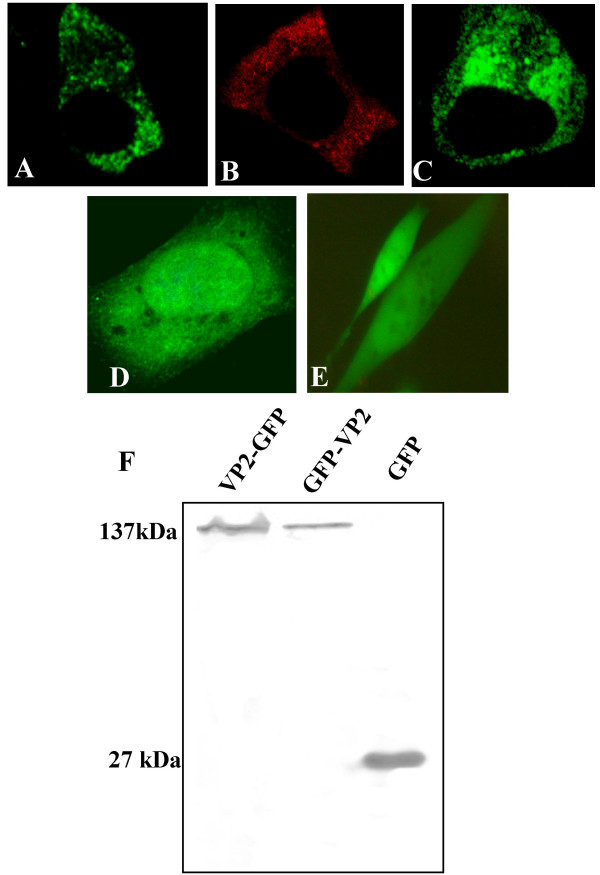
**Distribution of VP2 within infected and transfected Vero cells by fluorescence microscopy**. A) Cells infected with BTV-10, B-E) transfected cells expressing B) VP2, C) VP2-GFP, D) GFP-VP2 and E) GFP only. VP2 in A and B were detected with anti VP2 monoclonal antibody (rabbit) and either FITC (A) or TRITC (B) conjugated secondary antibody. C-E were visualised based on GFP fluorescence. Expression of full-length, tagged VP2 variants was confirmed by western blot using an anti-GFP antibody (F).

In order to assess the intracellular localisation of VP2 in virus infection, cells were first infected with BTV-10 and then VP2 detected by immunofluorescence microscopy. At 4 hours post infection VP2 was found to be restricted to punctuate areas within the cytoplasm (Fig. 2A). A similar distribution of VP2 was observed when cells were transfected with plasmids encoding VP2 or a VP2-GFP fusion protein where the GFP was located at the C-terminus of VP2 (Fig. 2B and 2C, respectively). However, fusion of GFP to the N-terminus of VP2 altered the subcellular localisation of the protein and resulted in a more diffuse pattern of fluorescence (Fig. 2D). Western blot analysis of the VP2 GFP fusion proteins confirmed that they were expressed in cells as full-length proteins (Fig. 2F).

### The N-terminus of VP2 is sufficient to direct sub-cellular localisation

**Figure 3:**
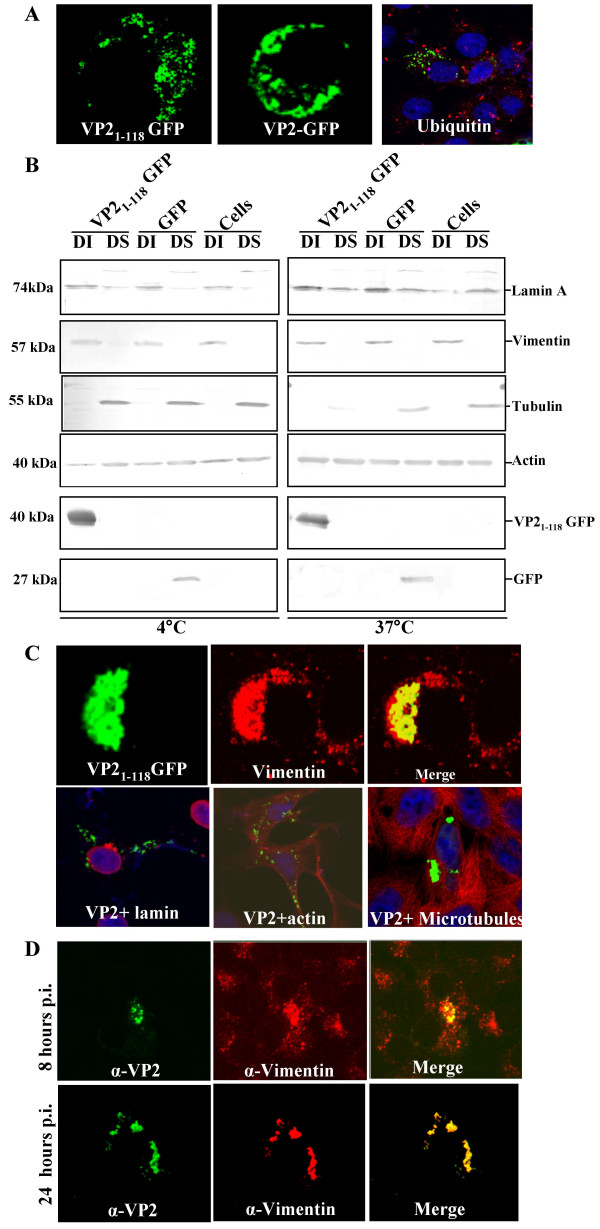
**VP2 segregates with vimentin in infected and transfected cells**. A) Distribution of VP2_1–118_GFP (left), VP2-GFP (centre), and VP2-GFP with ubiquitin (red, right) in transfected cells. Amino acids 1–118 give similar localisation to full-length protein. There is no evidence of co-localisation of ubuquitin with the punctuate distribution of VP2. B) Fractionation of untreated cells and cells transfected with plasmid expressing GFP or VP2_1–118_GFP. Cells were solubulised in the presence of 1% Triton X-100 and fractionated into detergent insoluble (DI) and detergent soluble (DS) fractions. Western blot was used to detect fractionation of the cytoskeletal marker proteins actin, tubulin and vimentin and the nuclear envelope marker lamin A. Fractionation of VP2_1–118_GFP and GFP was followed using anti-GFP antibody. VP2_1–118_GFP co-fractionated with vimentin but none of the other proteins. C) Imunofluorescence microscopy co-localisation of VP2_1–118_GFP with vimentin, lamin, actin and tubulin, as indicated. D) Distribution of vimentin (red) and untagged VP2 (green) in virus infected cells at 8 hours (top row) and 24 hours (bottom row) post infection. Note co-localisation of VP2 and vimentin even at early times post infection.

Since the fusion of GFP to the N-terminus but not the C-teriminus of VP2 altered its sub-cellular localisation we hypothesised that the N-terminus of the protein contained a signal necessary for intracellular localisation that was disrupted in the fusion protein. To test this possibility, we expressed an N-terminal fragment of VP2 consisting of the first 118 residues of the protein fused at its C-terminus to GFP (Fig. 3A, VP2_1–118_GFP). The distribution of this deletion mutant was indistinguishable from that of the full-length VP2-GFP protein (Fig. 3A, VP2 GFP), suggesting that it contained the signals necessary for the subcellular localisation of VP2. The punctuate pattern of VP2_1–118_GFP accumulation was distinct from what would be expected if the chimeric protein had been incorporated into aggresomes as there was no evidence of co-localisation with ubiquitin (Fig. 3A, ubiquitin).

### VP2 in virus infected cells and VP2_1–118_GFP associate with vimentin

Earlier electron microscopic studies of BTV infected cells have suggested that the virus associates with the cell membrane and with vimentin intermediate filaments [22,26]. In order to test where VP2_1–118_GFP was localised within cells, subcellular fractionation studies were carried out in the presence of detergents at both 4°C and 37°C (Fig. 3B). These studies were designed to separate soluble cytoplasmic proteins and lipid raft associated proteins from cytoskeletal and nuclear fractions of the cell. As expected, actin was present as both soluble and cytoskeletal associated insoluble forms under the conditions of the study. Tubulin and untagged GFP were present predominantly in the detergent soluble cytosolic fraction. The nuclear membrane protein lamin was present predominantly in the detergent insoluble fractions at both 4°C and 37°C, although at the higher temperature the proportion of solubulised lamin was increased. Vimentin and VP2 co-segregated in these fractionation studies, and were present only in the detergent insoluble fractions (Fig. 3B). Thus, fractionation studies supported association with VP2 with detergent insoluble cell fractions containing vimentin. Immunofluorescence was performed with each of the proteins in the cell fractionation studies in Vero cells expressing VP2_1–118_GFP. While there was no evidence that either actin, tubulin or lamin co-localised with the VP2 deletion mutant, all of the VP2_1–118_GFP was detected in areas of the cell rich in vimentin (Fig. 3C). Thus, in both cell fractionation and colocalisation studies VP2_1–118_GFP and vimentin were detected together.

In order to confirm the association between vimentin and full-length VP2, the distribution of vimentin and VP2 in virus infected cells was monitored by immunoflorescence. BTV infection of mammalian cells in tissue culture leads to rapid changes in cell morphology with apoptosis, which is triggered during virus entry [27]. Thus the distribution of VP2 and vimentin was assessed at early (8 hours) and late (24 hours) timepoints following virus infection (Fig. 3D). At 8 hours post infection vimentin was distributed throughout the cell but was also present in concentrated foci within the cytoplasm. At the same timepoint, VP2 was detected in concentrated foci within the cells. Intriguingly, the largest foci of VP2 co-localised with the largest foci of vimentin. All the VP2 in detected in virus infected cells was found associated with vimentin, although not all the vimentin at this timepoint was associated with VP2 (Fig. 3D). In contrast, at 24 hours post infection, by which time there are substantial virus-induced changes in cell morphology almost all the vimentin and VP2 present in the cell colocalised (Fig. 3D). Thus, data from virus infected cells confirm the VP2-vimentin association detected with the VP2_1–118_GFP deletion mutant.

### Deletion of amino acids 65–114 abolishes subcellular localisation of VP2_1–118_GFP

**Figure 4:**
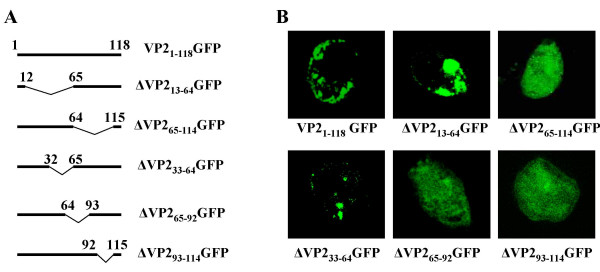
**Deletion anlysis of VP2_1–118_GFP**. A) Schematic showing the position of deletions generated in the VP2_1–118_GFP. B) Fluorescence images showing the distribution of VP2_1–118_GFP deletion mutants from A. Deletion of 65–92 and 93–114 was sufficient to abolish intracellular localisation of the N terminus of VP2.

Having established that VP2_1–118_GFP and full-length VP2 both colocalised with vimentin we went on to perform deletion mutagenesis of the VP2_1–118_GFP mutant in order to determine the regions of the protein critical to this interaction. Five deletions were introduced to the 1–118 region of VP2, to produce in-frame truncations of this region of the protein (Fig. 4A). Of these, deletion of the 49 amino acids between position 65 and 114 resulted in a diffuse distribution of the truncated protein (Fig. 4B). Further truncation of the protein, by introducing deletions from amino acids 65–92 or 93–114 gave no further resolution to the analysis, as both of these deletions abolished punctuate localisation of the VP2 protein (Fig. 4B). Deletions between amino acids 13 and 64 had no effect on the localisation of the VP2_1–118_GFP protein, suggesting that this region does not contribute to the subcellular localisation of the protein.

### Mutation of amino acids 70–72 of VP2 affects subcellular localisation but not multimerisation

**Figure 5:**
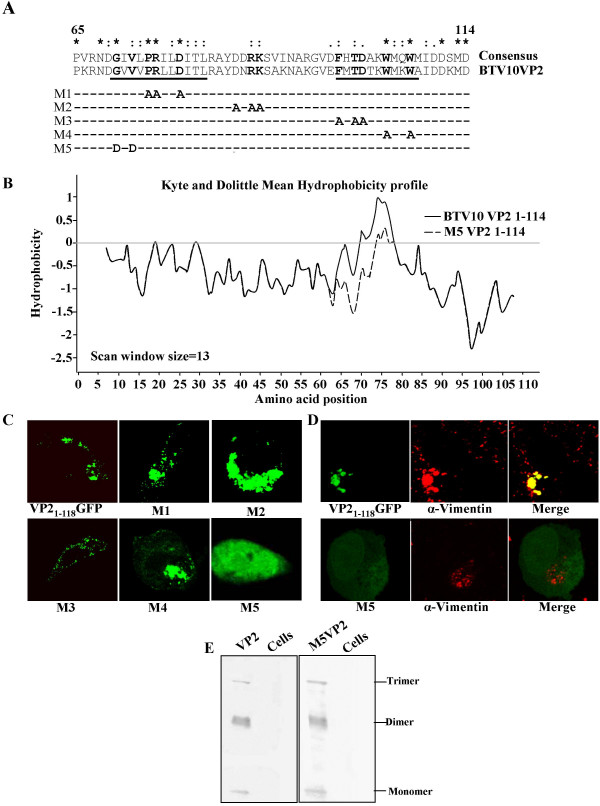
**Fine mapping of the influence of VP2 amino acids 65–114 on localisation**. A) Schematic showing the alignment of BTV-10 VP2 protein sequence to a consensus sequence generated from alignment of 13 BTV serotypes. Mutants generated (M1-M5) which disrupt conserved amino acid positions are indicated. Amino acids which are completely conserved or conserved in charge are indicated by (*) or (:) respectively. Weakly conserved amino acids are indicated by (.). Amino acids targeted for mutagenesis are shown in bold in the consensus and BTV-10 VP2 sequences. Sequences predicted *in silico *to form β-sheet are underlined. B) Kyte and Dolittle mean hydrophobicity profile[45], generated using a scan window size of 13 on BTV-10 VP2 1–114 and M5 VP2 1–114, as indicated. C) Fluorescence microscopy images of cells transfected with plasmid expressing VP2_1–118_GFP or M1-M5 as indicated. Intracellular localisation of VP2 is abolished in M5. D) Co-localisation of VP2_1–118_GFP and M5 with vimentin (red). Vimentin distribution is unaffected in cells expressing M5 but the VP2 mutant is found throughout the cell. E) Trimerisation of full-length VP2 carrying the M5 mutation. Western blot of non-denatured samples for unmodified (left panel) and M5 (right panel) VP2 variants. Note that trimerisation is unaffected in the M5 mutant.

The most likely reasons that deletions from 65–92 and 93–114 both resulted in diffuse localisation of the VP2 truncation mutant were that there were either multiple localisation signals, or the signal extended across the amino acid 92–93 junction, or these deletions had long-distance effects on folding of the remaining VP2 sequences. In order to resolve these possibilities we carried out site directed mutagenesis of conserved amino acids in this region of the protein. Sequence alignment of the sequence of the VP2 protein from 13 different BTV serotypes revealed that there were four amino acid regions within this sequence which were either completely conserved or very similar. These were the regions G70 to L81, D86 to K88, F98 to T100 and W104 to I109 (Fig. 5A). Five sets of mutations were generated; the first four were designed to mutate charged or conserved amino acids in each of the four conserved regions to alanine. The rationale for selecting the charged amino acids for mutation was that it was expected that these proteins would be more likely to be solvent exposed and thus available to interact with vimentin. The fifth mutation was to target the conserved GXV motif present at position 70–72. Since mutation of these amino acids to alanine would have been a very conservative change, and these residues are part of the only strongly hydrophobic region of the truncated protein, GVV was mutated to DVD. This mutation has the effect of altering the mean hydrophobicity of this region of the truncated VP2, such that the hydrophobic patch normally present in this region of protein has mean hydrophilic properties (Fig. 5B).

On expression, all of the four alanine mutations had similar punctate accumulation patterns to the unmodified VP2 sequence (Fig. 5C). In contrast, the mutation of the GVV to DVD at positions 70–72 resulted in a diffuse pattern of expression similar to that seen with the ΔVP2_65–92_GFP and ΔVP2_93–114_GFP deletion mutants. Vimentin targeted immunofluorescence with cells expressing the DVD (M5) mutant revealed that the association between vimentin and VP2 had been broken (Fig. 5D). Because the M5 mutation was designed to alter the hydrophobicity profile of the vimentin binding region of VP2 it was possible that the changes in accumulation seen were a result of global changes in the folding of the VP2 protein. In order to address this possibility, the M5 mutation was introduced into the full-length VP2 protein, and this protein expressed in insect cells using the baculovirus expression system. Unmodified VP2 multimerises to form a trimeric triskelion structure present on the outer surface of the mature virus particle [28]. By separating purified, unmodified VP2 under native conditions it is possible to detect monomeric, dimeric and trimeric forms of the protein (Fig. 5E). Separation of the full-length M5 VP2 mutant under the same conditions resulted in the formation of a similar proportion of each of the multimeric states of the VP2 protein as the unmodified protein. Thus the mutation that prevented the association of VP2 with vimentin did not prevent the formation of higher order multimers of the VP2 protein. This suggests that while it is possible that the local protein architecture was affected by the M5 mutation the overall folding of the protein was similar enough to the unmodified protein that multimerisation was unaffected.

### Pharmacological disruption of vimentin intermediate filaments inhibits virus release

**Figure 6:**
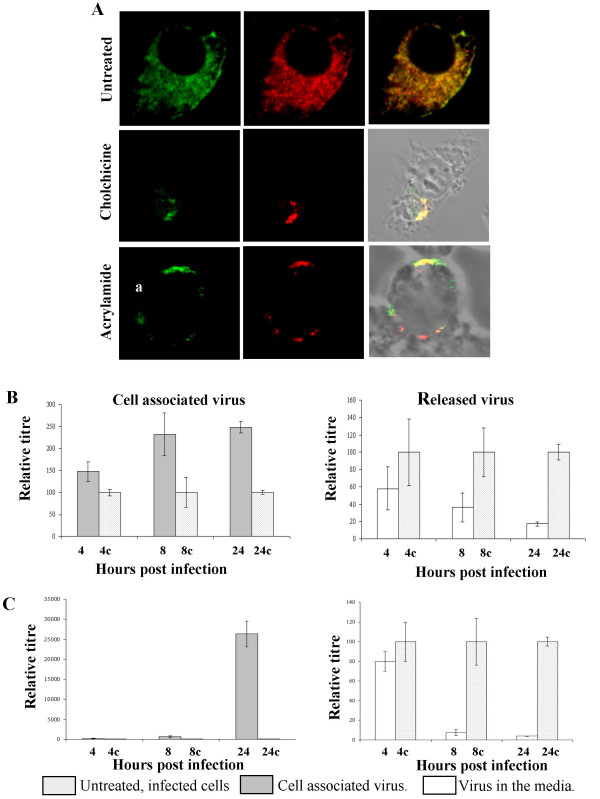
**Disrupting vimentin inhibits virus release in BTV infected cells**. A) Co-localization of untagged VP2 with vimentin in transfected cells. Top, untreated cells; Middle, cells treated with colchicine to disrupt microtubules; bottom, cells treated with acrylamide to disrupt vimentin. In each row VP2 and vimentin localisation are shown in green and red respectively. As expected, disruption of microtubules with colchicine causes a rearrangement in the localisation of vimentin. B) Effect of treating cells with colchicine on the release of virus from infected cells. Cells and culture medium were harvested at 4, 8 and 24 hours post infection with BTV-10 in untreated and colchicine (5 μg/ml) treated cells. Control samples are labelled 4c, 8c and 24c, respectively. The titre of cell associated and released virus for each timepoint normalised to 100% for untreated cells. Colchicine treatment resulted in a time dependent increase in the titre of cell associated virus and a time-dependent decrease in the titre of virus in the culture medium. D) As C but for cells treated with acrylamide (50 μM) to directly disrupt the vimentin network. Effect of acrylamide was similar but much faster and more dramatic than that observed by indirect disruption of vimentin through disruption of microtubules using colchicine. For C and D error bars indicate the standard error of three replicates of the experiments.

The vimentin intermediate filament network has a radial organisation, extending outwards from the cell centre. This localization partially overlaps with that of microtubules there is evidence that the two filament systems interact [29,30]. Indeed, vimentin structures move along microtubules [29,31-33]. Therefore, if the vimentin-VP2 interaction was important for BTV assembly and/or egress we predicted that pharmacological disruption of either microtubules or vimentin would result in changes of the amount of virus produced or the amount of virus released from infected cells. To test this possibility we carried out pharmacological experiments with colchicine and acrylamide which specifically disrupt the microtubule and vimentin intermediate filament networks respectively. Consistent with our previous experiments, in untreated cells vimentin was detected throughout the cytoplasm and this distribution was unaffected by the expression of full-length, untagged VP2. However, in the presence of colchicine, as expected, there was a redistribution of vimentin and VP2 towards the nucleus (Fig. 6A). This observation supports other observations that vimentin intermediate filaments are organised by the microtubule network [29,30]. The redistribution of vimentin was even more dramatic on the addition of acrylamide which resulted in a collapse of the intermediate filament network and a change in cell morphology to a more rounded shape (Fig. 6A). To test the effect of these changes in vimentin distribution on virus replication and release, virus was adsorbed to cells and then cells were treated with either colchicine (Fig. 6B) or acrylamide (Fig. 6B). Indirect disruption of vimentin with colchicine resulted in 2.5 times more cell associated virus and a five-fold reduction in the amount of the released virus detected compared to untreated control cells. Addition of acrylamide resulted in a 50 fold reduction in the amount of released virus and a 250 fold increase in the amount of cell associated virus at 24 hours post infection. These data suggest that disruption of vimentin either directly or indirectly inhibits the trafficking of mature virus particles out of virus infected cells.

## Discussion

The specific roles of the two proteins in the orbivirus outer capsid in the processes of virus attachment and membrane penetration have now been elucidated [4,9,10]. Intriguingly, one of these proteins, VP2, also plays a role in virus egress from infected cells as it binds a viral non-structural protein that acts as a molecular bridge between the virus particle and cellular export and release factors [24,25,34]. Thus, VP2 is important to both the infectivity and egress of BTV particles. The proteins that are required to form the transcriptionally active orbivirus core structure have all been localised to inclusion bodies within virus infected cells [17,35,36]. However, the outer capsid proteins do not localise to inclusions and therefore the site of assembly of mature virus particles before transport out of infected cells is unknown. Members of other genera in the *Reoviridae *cannot be used as a model for BTV in outer capsid assembly, as this information is either not available, or the outer capsid and assembly process is quite distinct. For example, unlike BTV, significant steps in the outer capsid assembly of rotavirus are sequestered in the endoplasmic reticulum [37]. Thus, in order to gain further insight into the assembly and intracellular trafficking of BTV, the localisation of VP2 in virus infected cells was followed using immunofluorescence microscopy. In virus infected cells VP2 was located in punctuate distribution throughout the cytoplasm even at quite early times post infection (Fig. 2). This distribution could be mimicked in cells transfected with plasmid expressing VP2 protein, full-length VP2 fused at its C-terminus to GFP, or the N-terminal 118 amino acids of VP2 fused to GFP (Fig. 2; Fig. 3). Furthermore, it was found that VP2 co-fractionated with the intermediate filament protein vimentin in fractionation studies, and co-localised with vimentin in immunofluorescence of VP2 in transfected and virus infected cells (Fig. 3). These data are consistent with earlier electron microscopy studies that have found mature virus particles in linear arrays along intermediate filaments in infected cells [26]. Furthermore they demonstrate for the first time that the determinant for BTV-vimentin association is the VP2 protein.

The vimentin associated localisation of VP2 in virus infected and VP2 transfected cells was quite distinct from the aggresome structure that has been noted following the overexpression of certain proteins. There was no evidence of the cage-like vimentin arrangement around VP2 which is observed with aggresomes [38,39]. Nor was there any evidence that ubiquitin co-localised with VP2 in transfected cells (Fig. 3).

Using a deletion mutagenesis strategy we were able to localise the vimentin localisation signal within VP2 to the region encompassing the amino acids 65–114 (Fig. 4). This was confirmed by systematic site-directed mutagenesis of conserved amino acids within this region to identify key residues in VP2-vimentin association. Of five sets of mutants, which changed a total of 13 conserved amino acids, only one, mutation of GVV to DVD at postions 70–72, prevented co-localisation of VP2 with vimentin (Fig. 5). This mutant was specifically designed to alter the hydrophobicity profile of the VP2 region from amino acids 63–80 to make it more hydrophilic. Thus, it was initially unclear whether the disruption of the association between VP2 and vimentin was due to global changes in the folding of the protein. However, when the DVD mutation was introduced into full-length VP2 the ability of the recombinant protein to assemble into trimeric VP2 structures was equivalent to that of unmodified protein. Thus, if there are changes to the structure of the protein induced by the DVD mutation, they occur at a local as opposed to a global level within the protein. These data indicate the DVD mutation either disrupts vimentin-VP2 association because the conserved GXV motif at position 70–72 is necessary for vimentin interaction or, like the deletions of aminio acids 65–92 or 93–114, mutation of this motif results in local rearrangement of the structure of VP2. At a primary sequence level, there is no apparent homology between this region of VP2 and vimentin, or any other known vimentin interacting protein. Therefore, in combination with the identity of the completely conserved amino acid (Glycine) and our mutagenesis data it is likely that VP2-vimentin interaction involving amino acids 65–114 is conformationally dependent.

Within the cell vimentin is emerging as a key player in intracellular trafficking. Vimentin structures have been shown to move along microtubules, and two candidate motors had been identified for this motility: conventional kinesin, and more recently, cytoplasmic dynein [29,31-33]. Significantly for VP2-vimentin interactions, the motility of vimentin intermediate filaments is principally from the nucleus towards the cell surface in BHK-21 cells [33]. Indeed, in this study when vimentin intermediate filaments were disrupted pharmacologically either directly, or indirectly by disrupting microtubules, there was a dramatic reduction in the amount of virus released from infected cells (Fig. 6). This reduction in release was not entirely due to a decrease in overall virus synthesis as, particularly in the case of acrylamide treatment, decrease in BTV release corresponded to an increase in cell associated virus (Fig. 6B). A plausible explanation for these observations would be that VP2-vimentin interaction is involved in the transport of mature virus particles. This hypothesis would correlate well with recent evidence that the viral non-strucutral protein NS3, which interacts with VP2 at its C-terminus [24], interacts at its N-terminus with the cellular release factor Tsg101 [25]. In this model, mature virus particles would be assembled from cores released from juxtanuclear inclusions, trafficked to the cell surface through vimentin association and released through the interaction of NS3 and Tsg101. Future work will focus on testing the validity of this model.

## Conclusion

Principal conclusions from this study are: i)The interaction that has been observed between mature BTV particles and vimentin intermediate filaments [26] is dependent on outer capsid protein VP2. ii) Amino acids 1–118 of VP2 are sufficient to determine vimentin association and within this region deletion of residues 65–114 or mutation of amino acids 70–72 to DVD is sufficient to abolish vimentin interaction. iii) Disruption of the vimentin intermediate filament network leads to an accumulation of intracellular virus particles and a reduction in virus release possibly because vimentin is involved in BTV trafficking to the cell surface.

## Methods

### Cells and viruses

A cell culture-adapted BTV serotype 10isolate (BTV-10; Colorado isolate) was used. The virus was propagated in Vero (African Green Monkey Kidney) cells and its titre was determined by plaque assay in BSR (Baby Hamster Kidney) cells. Vero and BSR cells were were incubated at 37°C in Dulbecco's modified Eagle's medium (Gibco BRL) containing 10% foetal calf serum (FCS), 100U penicillin/ml, and 100 μg streptomycin/ml (Sigma-Aldrich Chemical Co., St. Louis, Mo.). Recombinant baculovirus expressing bluetongue virus VP2 (AcMNPV-VP2) was propagated in *Spodoptera frugiperda *(*Sf21*) cells grown in suspension cultures at 28°C in TC100 medium (Sigma-Aldrich) supplemented with 10% foetal calf serum. Purification of recombinant VP2 was completed as described [4,40].

### Antibodies and pharmacological reagents

Monoclonal antibody against BTV-10 VP2 was a gift from N J. MacLachlan (School of Vet. Med., Davis, CA). Polyclonal antibodies against vimentin (goat), actin (goat) and GFP (rabbit) were purchased from Santa Cruz Biotech, CA. Polyclonal antibodies against ubiquitinin, tubulin and nuclear lamin were purchased from Abcam (Cambridge UK). Tetramethyl rhodamine isothiocyanate (TRITC) and fluorescein isothiocyanate (FITC)-conjugated secondary antibodies were purchased from Sigma-Aldrich. For pharmacological experiments, molecular grade acrylamide and cholchicine were purchased from Sigma-Aldrich.

### Construction of fusion proteins and VP2 mutants

Plasmids expressing VP2 were constructed by PCR amplifying the full-length L2 gene from BTV-10 and ligating it downstream of the Pol II or T7 RNA polymerase promoters in the pCAG-GS (The CABRI Consortium) or pCITE-2a vectors (Novagen), respectively.

To express the N-terminal GFP tagged VP2 fusion protein (GFP-VP2), GFP was amplified by PCR without its stop codon and inserted in-frame upstream of the VP2 protein. To express C-terminal GFP tagged VP2 (VP2-GFP) the normal VP2 stop codon was mutated to a restriction site using the QuickChange™ mutagenesis system (Stratagene, La Jolla, CA) and the GFP gene was excised from the pEGFP plasmid and ligated in-frame to the modified VP2 gene. VP2_1–118_GFP was produced by cloning EGFP as a blunt-end fragment from pEGFP (Clontech, Mountain View, CA) into a plasmid containing the full-length BTV-10 VP2 gene cut with *AflII*. For deletion analyses, site-directed mutagenesis was used to introduce unique restriction sites in VP2_1–118_GFP plasmid and internal deletions were introduced by cutting at the inserted restriction sites and religating the plasmid. Mutations were designed to introduce minimal changes to the amino acid sequence of the resulting deletion mutants. The QuickChange™ system (Stratagene) was used to generate amino acid substitution mutants in the VP2_1–118_GFP plasmid according to the manufacturer's protocol.

To express full-length VP2 with the DVD mutation at amino acids 70–72 the transfer vector pAcYM10.2 [40] expressing wild-type VP2 was mutated using the same primers and protocol as to introduce the mutation into the VP2_1–118_GFP plasmid. The orientation of constructs in all the plasmids was examined by restriction enzyme analysis, and the authenticity of each construct was confirmed by DNA sequencing using the ABI Prism^® ^Big Dye™Terminator cycle sequencing ready reaction kits (Applied Biosystems, Foster City, CA).

### Isolation of recombinant baculoviruses expressing mutant VP2 protein

Recombinant baculovirus expressing the DVD mutant for VP2 was produced using standard baculovirus recombination procedures as described [41]. Recombinant baculoviruses were plaque purified and propagated in *Sf*21 cells as described elsewhere [41].

### Expression of proteins from transfected plasmids and virus infection

Vero cells were plated in six well plates and plasmid DNA was transfected with Lipofectamine Plus™ transfection reagent (Invitrogen, Carlsbad, CA) following the manufacturer's instructions. Expression was analysed at 12–72 hours post-transfection as indicated in the text.

### Confocal microscopy

BTV infected or plasmid transfected cells were fixed on coverslips by incubation for 30 min at room temperature with 4% (w/v) paraformaldehyde in phosphate-buffered saline (PBS). Cover slips were then incubated for 15 min at room temperature with 1% Triton X in PBS (pH 7.5) and blocked for an hour with PBS containing 1% bovine serum albumin. Cells were incubated with primary antibodies diluted (1:100) in blocking buffer for 1 h, washed 5 times with PBS then incubated with appropriate secondary antibodies conjugated to TRITC (1:64) or FITC (1:128) prior to washing with PBS and mounting in Vectashield mounting media (Vector Laboratories, Burlingham, CA). Coverslips were examined with a Zeiss Axiovert 200 M laser-scanning microscope fitted with a helium-argon laser. Images were acquired and analysed using LSM 510 confocal software (Zeiss). For GFP imaging, cells grown on cover slips were transfected with the different GFP chimeras, washed and fixed at 12 hour intervals, from 0 to 48 hours post transfection as described previously.

### Isolation of Detergent Soluble (DS) and Insoluble (DI) fractions

Fractions were essentially prepared as described [42], with some minor modifications. In brief, 72 hours post transfection, cells were rinsed twice with ice-cold PBS, re-suspended and centrifuged at 1000 g for 5 minutes (Sanyo Micro Centaur, Jepson Bolton & Co Ltd, Watford, UK). The cell pellet was lysed for 30 min at 4°C in 0.2 ml of a 25 mM Tris-HCl (pH 7.5) buffer, containing 150 mM NaCl, 5 mM EDTA, and 1% Triton X-100, followed by 30 strokes in a dounce homogeniser. detergent soluble and insoluble fractions were separated by centrifugation for 15 minutes at 12,000 g (4°C) and resolved by SDS/10% Polyacrylamide gel electrophoresis (PAGE) [43]. In some experiments, cell lysis was carried out at 37°C instead of 4°C, and 1% Nonidet P-40 (NP40) was used instead of 1% Triton X-100 in the lysis buffer, as indicated in the text. SDS PAGE gels were stained with Coomassie brilliant blue as standard.

### Analysis of the oligomeric nature of VP2

*Sf*21 cell monolayers were infected with recombinant baculoviruses (pAcNPV.VP2 or pAcNPV.VP2M1) at a MOI of 3. Cells were harvested at 72 hours post infection, washed with PBS and lysed in 20 mM sodium phosphate buffer containing 150 mM NaCl [pH 7.5], 1% [v/v] Triton X and protease inhibitors (Sigma-Aldrich) at 4°C for 10 minutes. The lysate was clarified by centrifugation for 10 min at 10,000 rpm (Sanyo Micro Centaur, Jepson Bolton & Co Ltd, Watford, United Kingdom) and sample buffer added to a final concentration of (1% SDS, 15% glycerol, 10 mM Tris-HCl [pH 6.8], 0.02% [wt/vol] bromophenol blue) without 1% β-mercaptoethanol, without heating, and resolved by SDS-7% PAGE followed by western blotting.

### Western blot analysis

SDS-PAGE separated proteins were transferred onto a Hybond enhanced chemiluminescence nitrocellulose membrane (GE Healthcare, Uppsala Sweden) as described previously [44] and were probed with appropriate antibodies to GFP, vimentin, tubulin, lamin and actin. Subsequently, the blots were incubated with alkaline phosphatase conjugated secondary antibodies and developed with BCIP-NBT substrate (Sigma-Aldrich).

### Treatment of the cells with cytoskeleton inhibitors

Cells were washed 48 hours post transfection and incubated in medium with 50 μM of acrylamide (24 hours) or 5 μg/ml of colchicine (2 hours). They were then processed for immunofluorescence staining as described above. To examine the effect of the inhibitors on viral yield at different time points, the cells were first infected as described previously and then pharmacologically treated. Cell supernatant and cell pellets were collected and were subjected to three freeze/thaw cycles to disrupt cells and to release virus. All samples were collected, the viral titer of each sample was determined in triplicate by virus plaque assay in BSR cell monolayer cultures. Infectivity titers were calculated as log10 pfu/ml, and the mean and standard error of the reduction mediated by each inhibitor was calculated (Sigma-AldrichPlot 2000, SYSTAT Software Inc.). All the pharmacological treatments were performed in duplicate and the plaque assay in triplicate.

## Competing interests

The author(s) declare that they have no competing interests.

## Authors' contributions

BB carried out the confocal microscopy, mutagenesis and fractionation studies. RN conceived the study, made the initial observations on the localisation of GFP-tagged VP2 and wrote a draft of the manuscript. PR contributed ideas to the design and co-ordination of the study and secured funding for the project. All authors read and approved the final manuscript.
